# Carvedilol Improves Inflammatory Response, Oxidative Stress and Fibrosis in the Alcohol-Induced Liver Injury in Rats by Regulating Kuppfer Cells and Hepatic Stellate Cells

**DOI:** 10.1371/journal.pone.0148868

**Published:** 2016-02-18

**Authors:** Raimundo Fernandes de Araújo Júnior, Vinícius Barreto Garcia, Renata Ferreira de Carvalho Leitão, Gerly Anne de Castro Brito, Emilio de Castro Miguel, Paulo Marcos Matta Guedes, Aurigena Antunes de Araújo

**Affiliations:** 1 Postgraduate Program in Health Science, UFRN, Natal, RN, Brazil; 2 Postgraduate Program in Functional and Structural Biology/Department of Morphology/UFRN, Natal, RN, Brazil; 3 Department of Morphology/Postgraduate Program in Morphology/UFC, Fortaleza, CE, Brazil; 4 Department of Physical/Analytical Center/UFC, Fortaleza, CE, Brazil; 5 Department of Microbiology and Parasitology, UFRN, Natal, RN, Brazil; 6 Department of Biophysics and Pharmacology, UFRN, Postgraduate Programs in Public Health and Pharmaceutical Science, Natal, RN, Brazil; University of Navarra School of Medicine and Center for Applied Medical Research (CIMA), SPAIN

## Abstract

**Aim:**

To evaluate the anti-inflammatory, anti-oxidant and antifibrotic effects of carvedilol (CARV) in rats with ethanol-induced liver injury.

**Methods:**

Liver injury was induced by gavage administration of alcohol (7 g/kg) for 28 consecutive days. Eighty Wistar rats were pretreated with oral CARV at 1, 3, or 5 mg/kg or with saline 1 h before exposure to alcohol. Liver homogenates were assayed for interleukin (IL)-1β, IL-10, and tumor necrosis factor (TNF)-α level as well as for myeloperoxidase (MPO) activity and malonyldialdehyde (MDA) and glutathione (GSH) levels. Serum aspartate aminotransferase (AST) activity and liver triglyceride (TG) levels were also assayed. Immunohistochemical analyses of cyclooxygenase 2 (COX-2), receptor activator of nuclear factor kappa-B/ligand (RANK/RANKL), suppressor of cytokine signalling (SOCS1), the Kupffer cell marker IBA-1 (ionized calcium-binding adaptor molecule 1), intercellular adhesion molecule 1 (ICAM-1), superoxide dismutase (SOD-1), and glutathione peroxidase (GPx-1) expression were performed. Confocal microscopy analysis of IL-1β and NF-κB expression and real-time quantitative PCR analysis for TNFα, PCI, PCIII, and NF-κB were performed.

**Results:**

CARV treatment (5 mg/kg) during the alcohol exposure protocol was associated with reduced steatosis, hepatic cord degeneration, fibrosis and necrosis, as well as reduced levels of AST (*p* < 0.01), ALT (*p* < 0.01), TG (*p* < 0.001), MPO (*p* < 0.001), MDA (*p* < 0.05), and proinflammatory cytokines (IL-1β and TNF-α, both *p* < 0.05), and increased levels of the anti-inflammatory cytokine IL-10 (*p* < 0.001) and GSH (*p* < 0.05), compared to the alcohol-only group. Treatment with CARV 5 mg/kg also reduced expression levels of COX-2, RANK, RANKL, IBA-1, and ICAM-1 (all *p* < 0.05), while increasing expression of SOCS1, SOD-1, and GPx-1 (all *p* < 0.05) and decreasing expression of IL-1β and NF-κB (both, *p* < 0.05). Real-time quantitative PCR analysis showed that mRNA production of TNF-α, procollagen type I (PCI), procollagen type III (PCIII), and NF-κB were decreased in the alcohol-CARV 5 mg/kg group relative to the alcohol-only group.

**Conclusions:**

CARV can reduce the stress oxidative, inflammatory response and fibrosis in ethanol-induced liver injury in a rat model by downregulating signalling of Kuppfer cells and hepatic stellate cells (HSCs) through suppression of inflammatory cytokines.

## Introduction

Alcohol-induced liver disease (ALD) has a wide range of presentations, ranging from simple steatosis to cirrhosis and hepatocellular carcinoma. ALD continues to be a major health issue worldwide [1]. Chronic ethanol administration to rodents leads to numerous hepatic changes, including steatosis, hepatocellular necrosis, inflammatory cell infiltration, terminal hepatic venular sclerosis, proliferation of the smooth endoplasmic reticulum, and mitochondrial aberrations. All of these changes also occur during the early phases of human ALD, demonstrating the relevance of rodent ALD models [2].

Many histological abnormalities associated with ethanol-induced steatosis are particularly prevalent in the perivenular region of the liver lobules, due to the lower oxygen concentration in this region and the increased susceptibility of perivenous cells to ischemic necrosis. Low-oxygen conditions that are sufficiently hypoxic to damage hepatocytes may develop in this area after alcohol consumption [3]. Another factor that may contribute to ethanol-induced injury in the perivenular region is the high regional expression of CYP2E1, which has been associated with the production of potentially harmful oxygen radicals [4]. Furthermore, hepatocytes in the perivenous area contain relatively low levels of antioxidant factors [*e*.*g*., glutathione (GSH)] and antioxidant enzymes [*e*.*g*., glutathione peroxidase (GPx-1)] [5].

The most convincing data indicating that oxidative stress contributes to alcohol-induced liver injury come from studies using the intragastric infusion model of alcohol administration. In these studies, alcohol-induced liver injury was associated with enhanced lipid peroxidation, protein carbonyl formation, formation of the 1-hydroxyl ethyl radical, formation of lipid radicals, and decreases in hepatic antioxidant defences, especially GSH. Furthermore, the small Kupffer cells in the perivenous area produce high levels of cytotoxicity-associated cytokines [6].

Alcohol intake increases intestinal permeability to various substances, including bacterial endotoxins, such as lipopolysaccharide [7]. Lipopolysaccharide sensitizes Kupffer cells by binding to the CD14 receptor, thereby activating nuclear factor (NF)-κB, which increases the transcription of proinflammatory cytokines, such as tumor necrosis factor (TNF)-α, interleukin (IL)-6, and transforming growth factor (TGF)-β. TNF-α and IL-6 play important roles in cholestasis and the synthesis of acute-phase proteins. Meanwhile, TGF-β plays a central role in fibrogenesis through the activation of hepatic stellate cells (HSCs), a process of fibrosis that is associated with necro-inflammation and apoptosis, and which leads to the progression of liver disease and, ultimately, cirrhosis.

The suppressor of cytokine signalling (SOCS) family consists of SOCS1–7 and cytokine-inducible SH2-containing protein. Members of this protein family mediate negative feedback regulation of the cytokine-induced Janus kinase (JAK)/signal transducer and activator of transcription (STAT) signal transduction pathway [8]. SOCS1 has been reported to inhibit inflammation [9], and cytokines whose expression is induced by inflammation have been shown to upregulate SOCS1 expression [10].

Under normal conditions, HSCs are quiescent and produce only small amounts of extracellular membrane (ECM) constituents, such as laminin and collagen type IV, which are essential components of basement membranes [11]. Upon exposure to soluble factors from damaged hepatocytes or activated Kupffer cells, HSCs reduce their lipid content (retinyl palmitate), and undergo a morphological transition to myofibroblast-like cells (11). Activated HSCs then produce large amounts of ECM components, including collagen I, in an accelerated fashion, triggering a fibrogenic response [12].

Carvedilol (CARV) blocks sympathetic neural activation via antagonism of β1-, β2-, and α1-adrenoreceptors. CARV has been shown to provide greater cardiovascular benefits than traditional β-blockers in both humans and animals, and these benefits have been attributed to its antioxidant, anti-inflammatory, and antifibrotic properties [13,14]. CARV has been shown to contribute to the attenuation of alcoholic fatty liver disease development in rats and appears to improve ethanol-induced liver injury by modifying the interaction between oxidative stress and sympathetic hyperactivity [15]. The antifibrotic effects of CARV have been associated with the amelioration of oxidative stress in carbon tetrachloride-induced hepatotoxicity model [16] and suppression of HSC-derived lipogenesis- and fibrogenesis-related mediators [17].

The goal of this study was to characterize the anti-inflammatory activity of CARV in a rat model of ethanol-induced liver injury through an analysis of markers of the alcohol-induced inflammatory process. We also examined procollagen type I (PCI) and procollagen type III (PCIII) levels in ethanol-injured livers, with or without CARV, because HSC cells undergo an activation process in response to liver injury, in which they produce a fibrotic matrix rich in PCI and PCIII.

## Materials and Methods

### Chemicals

Ethanol was purchased from LIBBs Farmacêutica Ltda, São Paulo, Brazil. Carvedilol (Ictus 6,25mg, Biolab Sanus Farmacêutica ltda, São Paulo, Brazil), O-Dianisine Sigma (São Paulo, Brazil), antibodies (Santa Cruz Biotechnology, INTERPRISE, Brazil): COX-2; RANK; RANKL; SOCS-1, Streptavidin-HRP-conjugated secondary antibody (Biocare Medical, Concord, CA, USA). TrekAvidin-HRP Label + Kit from Biocare Medical, Dako, USA. IL-1β, IL-10, TNF-α ELISA kit (R&D Systems, Minneapolis, MN, *USA*).

### Preparation and administration of ethanol

7g per kg body weight of 30% v/v ethanol solution was used as chronic dose in this experiment. 30g absolute ethanol was dissolved in distilled water and made up to 100ml. 6,2ml of the solution was daily administered for four weeks to each rat treated with ethanol.

### Animal preparation

Experiments were performed on male Wistar albino rats weighing between 250 and 300 g, purchased from Bioterio Department of Biophysical and Pharmacology. Animals were housed in a temperature and humidity controlled environment under a 12-h light/dark cycle (lights on at 6 AM). Food and water were available *ad libitum*. The animals used in the experiments originated from the Department of Biophysics and Pharmacology. The animals were housed individually in polypropylene cages measuring 41 x 34 x 16 cm Autoclaved. Various signals of the health of the animals were monitored, including: coat condition, response to stimuli, faeces and urine. Only those animals in perfect health were kept in the experiment. Standard diet (Basic Composition: Soybean meal, dextrin, rice husks, wheat bran, rice bran, meat meal, fish meal, sodium chloride, magnesium oxide, iron sulfate, copper sulfate, manganese monoxide, zinc oxide, calcium iodate, cobalt sulphate, sodium selenite, vitamin A, vitaminD3, vitamin E, vitaminK3, vitamin B1, vitaminB2, niacin, pantothenic acid, vitaminB6, folic acid, biotin, vitamin B12, choline chloride, lysine, methionine, propionic acid, *Agrobacteriumtumefaciens*, and *Bacillusthuringiensis;* Presence/Evialis do Brasil Nutrição Animal LTDA, São Paulo) and water source (bottled). The National Institutes of Health Guidelines for the Care and Use of Laboratory Animals were followed. All efforts were made to minimize the number of animals used and their suffering degree. Animals were sacrificied on by an overdose of anaesthesia with 2% thiopental (80 mg/kg, i.p.). After death a cardiac puncture was performed. The sacrificed animals were kept in -20°C freezer. Animals were monitored after induction of ethanol induced liver injury for once week once week during for 28 days. There was not any incidental mortality as a result of liver injury. This research and methods used in this investigation were approved by by the Animal Ethics Committee–CEUA- of the Universidade Federal do Rio Grande do Norte (approval number: 053/2013).

### Induction of ethanol-induced liver injury

Eighty rats were randomly divided into eight groups (five animals per group, duplicate groups). The vehicle control group received normal saline orally by gastric gavage and i.p saline (0.9% NaCl) for 28 days. The positive control group received saline orally by gastric gavage and after 01 hour ethanol (7 g/kg) by gastric gavage too for 28 days.

Three groups received oral CARV at 1, 3 and 5 mg/kg, respectively, by gastric gavage and after 01 hour saline (0.9% NaCl) by gastric gavage too for 28 days. Three additional groups received oral CARV at 1, 3 and 5 mg/kg by gastric gavage too, respectively, by gastric gavage and after 01 hour ethanol (7 g/kg) for 28 days. Animals were euthanized on the twenty-ninth day with (80 mg/kg, i.p.) 2% thiopental (Cristália, São Paulo, Brazil).

Following euthanasia, a cardiac puncture was performed and blood samples were taken for leukogram and biochemical analyses. The livers of the rats were frozen at -80°C for analyses of cytokine and myeloperoxidase (MPO) levels. Livers were immersed in 10% buffered formalin for histopathological analysis.

### Myeloperoxidase (MPO) Activity

The extent of neutrophil accumulation in the Liver samples was measured by assaying MPO activity. Liver samples were harvested as described above and stored at −70°C until required for assay. After homogenisation and centrifugation (2000 × *g* for 20 min), MPO activity was determined by a previously described colorimetric method [18]. Results are reported as units of MPO per gram of tissue.

### Malonyldialdehyde (MDA) assay

Malonyldialdehyde (MDA) is an end product of lipid peroxidation. To quantify the increase in free radicals in liver sample, MDA content was measured via the assay described by Esterbauer and Cheeseman [19]. Liver samples were suspended in buffer Tris HCl 1:5 (w/v) and minced with scissors for 15 sec on an ice-cold plate. The resulting suspension was homogenised for 2 min with an automatic Potter homogenizer and centrifuged at 2500 × g at 4°C for 10 min. The supernatants were assayed to determine MDA content. The results are expressed as nanomoles of MDA per gram of tissue.

### Glutathione (GSH) levels

GSH levels in liver tissues were measured to antioxidant. GSH content was measured via the assay described by Costa et al [20]. Liver samples (5 per group) were stored at 70°C until use. Liver tissue homogenates (0.25 mL of a 5% tissue solution prepared in 0.02 M EDTA) were added to 320 mL of distilled water and 80 mL of 50% TCA. Samples were centrifuged at 3000 rpm for 15 minutes at 4°C. The supernatant (400 mL) was added to 800 mL of 0.4 M Tris buffer at pH 8.9 and 20 μL of 0.01 M DTNB. The absorbance of each sample was measured at 420 nm, and the results were reported as units of GSH per milligram of tissue.

### IL-1β, IL-10, and TNF-α assay

Liver samples (three samples per group) were stored at −70°C until use. The tissue was homogenised and processed as described by Safieh-Garabedian, et al (1995) [21]. Levels of IL-1β (detection range: 62.5–4000 pg/mL; sensitivity or lower limit of detection [LLD]: 12.5 ng/mL of recombinant mouse IL-1β), IL-10 (detection range: 62.5–4000 pg/mL; sensitivity or LLD: 12.5 ng/mL of recombinant mouse IL-10) and TNF-α (detection range: 62.5–4000 pg/mL; sensitivity or LLD: 50 ng/mL of recombinant mouse TNF-α) in the intestinal samples were determined with a commercial ELISA kit (R&D Systems, Minneapolis, MN, *USA*), as described previously. All samples were within the wavelength used in UV-VIS spectrophotometry (absorbance measured at 490 nm).

### Light microscopy studies and biochemical analysis

The livers were excised quickly and washed with cold isotonic saline. Each segment was weighed and cut longitudinally. Three sections of liver (five animals per group) were analyzed. The specimens were fixed in 10% neutral buffered formalin, dehydrated and embedded in paraffin. Sections of 5 μm thickness were obtained for haematoxylin–eosin staining (H&E) and examined by light microscopy (40x, Olympus BX50, Morphology Department/UFRN). Liver pathology was scored as follows: steatosis (the percentage of liver cells containing fat): <25% = 1, < 50% = 2, <75% = 3, >75% = 4.; inflammation and necrosis: 1 focus per low-power field; 2 or more foci. Pathology was scored in a blinded manner by one of the authors and by an outside expert in rodent liver pathology. Fat accumulation causes ballooning of hepatocytes and narrowing of the sinusoidal space. This could affect the number of hepatocytes and sinusoidal space in each field; therefore, the number of hepatocytes also was counted and the number of neutrophils was expressed per 100 hepatocytes [22,23]. The mean values were used for statistical analysis.

Histological sections were stained using picrosirius red staining kit (1% Sirius red in saturated picric acid; EasyPath, Indaiatuba, Brazil) for 24 h, or hematoxylin and eosin (Easypath) and examined under light microscopy (Nikon Eclipse 2000 equipped with Nikon DS-Fi2; Nikon Corporation, Tokyo, Japan).

For the purpose of Quantitative analysis the collagen content, randomly sampled two hundred light microscope images (200X) per liver specimen, including large centrilobular veins and large portal tracts (≥150 mm) were analyzed. About 20 polarized light microscopy images using an Olympus BX60 microscope (Olympus, Tokyo, Japan) (200X) per specimen were captured and analyzed using a color threshold detection system developed in ImageJ (National Institutes of Health). Known positive and negative controls were included in each batch of samples. Tissue reactivity in all groups (negative control, alcohol group and alcohol carv 5) was assessed. Values are expressed as percentage of positive area. Contrast index measurements were obtained from selected area x 100/ total area positioned across the regions of interest (three samples per animal). Moreover, hepatic fibrosis was quantified using Ishak scoring system^:^ level 1 indicating the absence of fibrosis; level 2 indicated enlargement of portal area; level 3 was assigned to fibrous expansion of most portal areas; level 4 was assigned to lobules with fibrous expansion of most portal areas with occasional portal to portal bridging; level 5 was assigned to lobules with fibrous expansion of most portal areas with marked bridging (portal to portal and portal to central); level 6 was assigned to lobules with marked bridging (portal to portal and portal to central) with occasional nodules (incomplete cirrhosis); level 7 was observed Cirrhosis in the lobules [24].

Blood samples collected from the rat tail at the end of the pair-feeding were centrifuged at 3000 *g* for 10 min, and resultant supernatants were used to measure the blood alanine aminotransferase level (ALT, U/L) to evaluate the ethanol-induced liver injury. Liver cytosolic protein solution was used to measure the hepatic triglyceride concentration (mg/g total liver protein, % of control) as a marker of the ethanol-induced lipid surplus in the liver. The levels of ALT and hepatic triglyceride were measured with an automatic analyzer (FDC4000; Fuji Medical Systems, Tokyo, Japan). The values are given as mean 1 SD of five animals.

### Immunohistochemical staining of COX-2, RANK, RANKL, IBA-1, ICAM-I, SOCS-1, SOD-1 and GPx-1

Thin sections of liver (4 μm) were obtained from each group (negative control, alcohol and alcohol CARV 5 mg/kg) with a microtome and transferred to gelatine-coated slides. Each tissue section was then deparaffinised and rehydrated. The liver tissue slices were washed with 0.3% Triton X-100 in phosphate buffer (PB) and quenched with endogenous peroxidase (3% hydrogen peroxide). Tissue sections were incubated overnight at 4°C with primary antibodies (Santa Cruz Biotechnology, INTERPRISE, Brazil) against COX-2, RANK, RANKL, SOCS-1, SOD-1 GPx-1 and primary antibodies (Spring-ABCAM, USA) against IBA-1 and ICAM-I. Dilution tests (3 dilutions) were performed with all antibodies to identify the 1:600, 1:800, 1:1000, 1:500, 1:800, 1:600 and 1:300, 1:500, dilutions as appropriate, respectively. Slices were washed with phosphate buffer and incubated with a streptavidin/HRP-conjugated secondary antibody (Biocare Medical, Concord, CA, USA) for 30 minutes. Immunoreactivity to the various proteins was visualised with a colorimetric-based detection kit following the protocol provided by the manufacturer (TrekAvidin-HRP Label + Kit from Biocare Medical, Dako, USA). Sections were counter-stained with hematoxylin. Known positive controls and negative controls were included in each set of samples. Planimetry microscopy (Nikon E200 LED, Morphology Department/UFRN) with a high-power objective (40×) was utilised to score the intensity of cell immunostaining: 1 = absence of positive cells; 2 = small number of positive cells or isolated cells; 3 = moderate number of positive cells; and 4 = large number of positive cells. Labelling intensity was evaluated by two previously trained examiners in a double-blind fashion. Three tissue sections per animal (five animals per group) were evaluated.

### Confocal immunofluorescence

Three tissue sections from each animal (5 animals per group) were deparaffinized in xylene and washed in a series of concentrations of ethanol and PBS. Antigen retrieval was performed by placing the sections in a 10 mM sodium citrate with 0.05% Tween 20 for 40 minutes at 95°C. Autofluorescence background noise was reduced by incubating the sections in 0.1% Sudan black in 70% ethanol for 40 min at Room Temperature (RT). The sections were incubated overnight with rabbit anti-IL-1β and NF-κB primary antibody (1:200 and 1:100, respectively, in blocking solution/1% normal goat serum; ABCAM, USA and Santa Cruz Biotechnology, USA, respectively) at 4°C, washed three times in PBS/0.2% triton X-100 for 5 min and incubated with Alexa Fluor 488- conjugated goat anti-rabbit secondary antibody (1:500 in BSA 1%) and DAPI nuclear counterstain (SIGMA, USA). Finally, the sections were mounted with Vectashield medium.

Fluorescent images were obtained on a Carl Zeiss Laser Scanning Microscope (LSM 710, 20× objective, Carl Zeiss, Jena, Germany, Brazil). Known positive and negative controls were included in each batch of samples. Tissue reactivity in all groups (negative control, alcohol, and alcohol carv 5) was assessed by computerised densitometry analysis of digital images captured with the aforementioned confocal immunofluorescence microscope. Average densitometric values were calculated in Image J software (http://rsb.info.nih.gov/ij/). Contrast index measurements were obtained from the formula (selected area × 100)/total area after removal of background in regions of interest (three samples per animal).

### RT-PCR quantitation of TNFα, PCI, PCIII, and NF-κB gene expression

Total RNA was extracted from liver tissue with TRIzol reagent (Invitrogen Co. USA) and the SV Total RNA Isolation System (Promega, Madison, WI). First-strand cDNA was synthesized from 1 μg of total RNA with the ImProm-IITM Reverse Transcriptase System for RT-PCR (Promega) according to the manufacturer’s protocol. Real-time quantitative PCR analyses of TNF-α, PCI, PCIII, and GAPDH mRNAs were performed with SYBR Green Mix in the Applied Biosystems® 7500 FAST system (Applied Biosystems, Foster City, CA), according to a standard protocol with the following primers: *GAPDH* (forward: 5’ AAC TTT GGC ATC GTG GAA GG 3’; reverse: 5’ GTG GAT GCA GGG ATG ATG TTC 3’, annealing primer temperature: 60°C), *TNFα* (forward: 5’ AGT CCG GGC AGG TCT ACT TT3’; reverse: 5’ TTC AGC GTC TCG TGT GTT TC 3’, annealing primer temperature: 56.5°C), *PCI* (forward:5’ CAG GGA GTA AGG GAC ACG AA 3’; reverse: 5’TCC CAC AGC AGT TAG GAA CC 3’, annealing primer temperature: 56.8°C), *PCIII* (forward: 5’ ATG GTG GCT TTC AGT TCA GC 3’; reverse: 5’ TGG GGT TTC AGA GAG TTT GG 3’, annealing primer temperature: 55.2°C), and *NF-κB* (forward: 5’ TCT GCT TCC AGG TGA CAG TG 3; reverse: 5’ ATC TTG AGC TCG GCA GTG TT 3’, annealing primer temperature: 55.2°C. The standard PCR conditions were as follow: 50°C for 2 min and 95°C for 10 min, followed by forty 30-s cycles at 94°C, a variable annealing primer temperature for 30 s, and 72°C for 1 min. The experiments were performed in triplicate and repeated at least three times. Mean Ct values were used to calculate the relative expression levels of the target genes for the experimental groups, relative to those in the negative control group; expression data were normalized relative to the housekeeping gene *GAPDH* using the 2–ΔΔCt formula.

## Results

### Animals’ health status

No animals died during the experiment; thus, the group numbers did not vary. Body weights of the animals were similar across the experimental groups (250–300 g throughout the experiment). Coat health and faeces control also did not differ between the groups throughout the experimental period. The alcohol-administered groups showed growing impairments in ambulation and spontaneous motor activity in the open-field activity test (Insight, São Paulo, Brazil), relative to no-alcohol controls, at the end of each week (data not shown).

### Effects of CARV on MPO activity and on MDA and GSH levels

**Fig 1:**
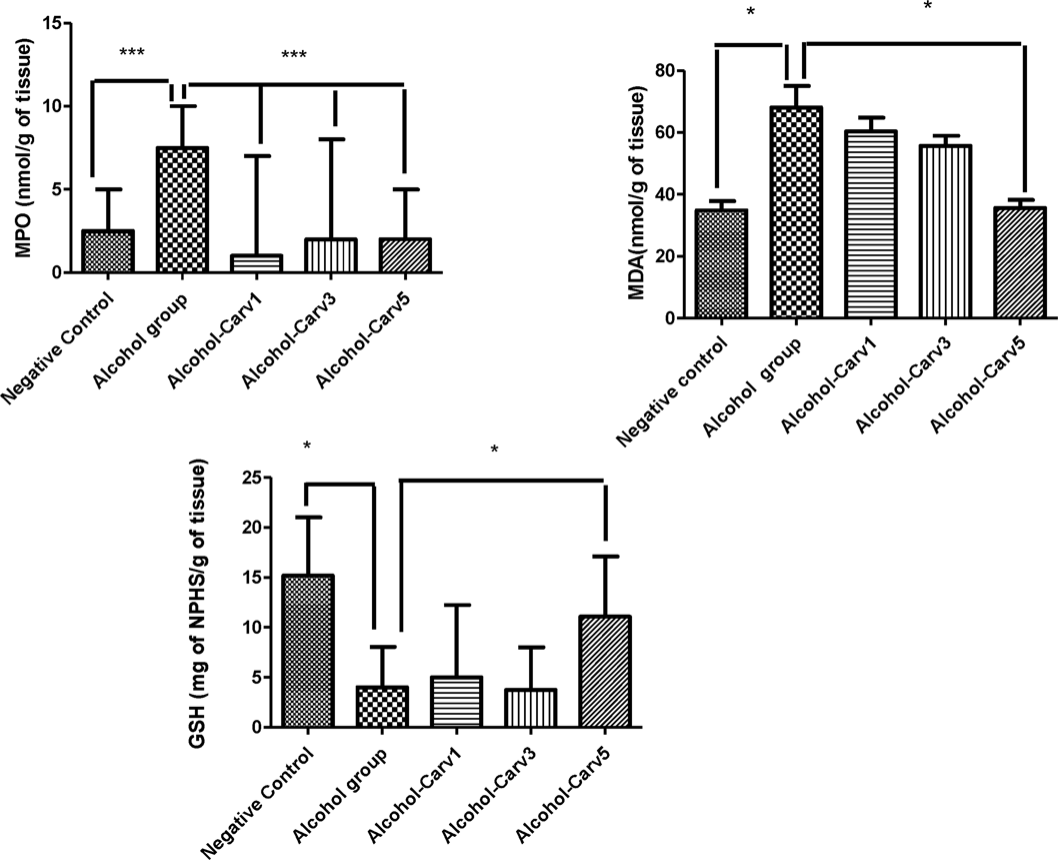
CARV modulates MPO activity, MDA activity, and GSH levels in the livers of rats with alcohol-induced liver injury. Experiments were conducted in duplicate. *p < 0.05; ***p < 0.001.

Livers from the alcohol-only treatment group had significantly greater MPO activity than livers harvested from the negative control group (*p* < 0.001), and this increase was attenuated in the groups that received alcohol with CARV (1 mg/kg, 3 mg/Kg, or 5 mg/kg). The high-dose CARV treatment (5 mg/kg) increased GSH levels (*p* < 0.05 *vs*. alcohol-only; Fig 1). Levels of MDA were increased significantly in the alcohol-only group livers compared to levels in livers from the negative control group (*p* < 0.05), and this increase was blocked in livers from animals given high-dose CARV (5 mg/kg; *p* < 0.05 *vs*. alcohol-only; Fig 1).

### Effect of CARV treatment on inflammation and IL-1β, IL-10, and TNF-α level

**Fig 2:**
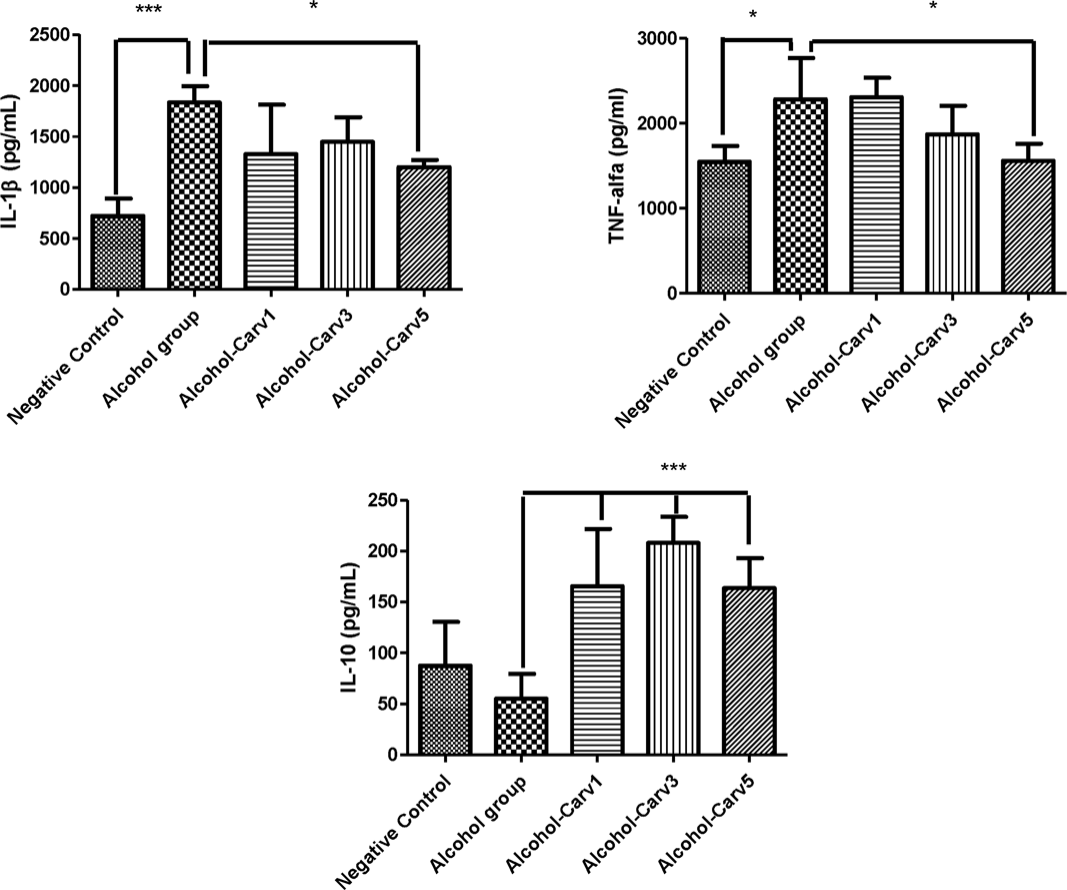
CARV counters alcohol-mediated effects on cytokine production in rats with alcohol-induced liver injury. Alcohol treatment elevated IL-1β (***p < 0.001) and TNF-α (**p < 0.01) levels, but reduced IL-10 levels (**p < 0.01), compared to the negative control group. CARV administration had a dose-dependent, alcohol-countering effect on IL-10 (5 mg/kg ***p < 0.001); only the 5 mg/kg dose of CARV had significant alcohol-countering effects on IL-1β (*p < 0.05) and TNF-α (5 mg/kg **p < 0.01) levels. Experiments were conducted in duplicate.

Alcohol administration elevated IL-1β (*p* < 0.001) and TNF-α (*p* < 0.01) levels, but reduced levels of IL-10 (p < 0.01), compared to levels in the negative control group. These alcohol-induced effects could be reversed with CARV treatment. Specifically, IL-1β and TNF-α level in the alcohol-CARV 5 mg/kg group were lower than in the alcohol only group (*p* < 0.05 and *p* < 0.01, respectively). Furthermore, IL-10 levels were higher in all three CARV dose groups (1 mg/kg, 3 mg/kg, 5 mg/kg) than in the alcohol-only group (all *p* < 0.001, Fig 2).

### Histology

**Fig 3:**
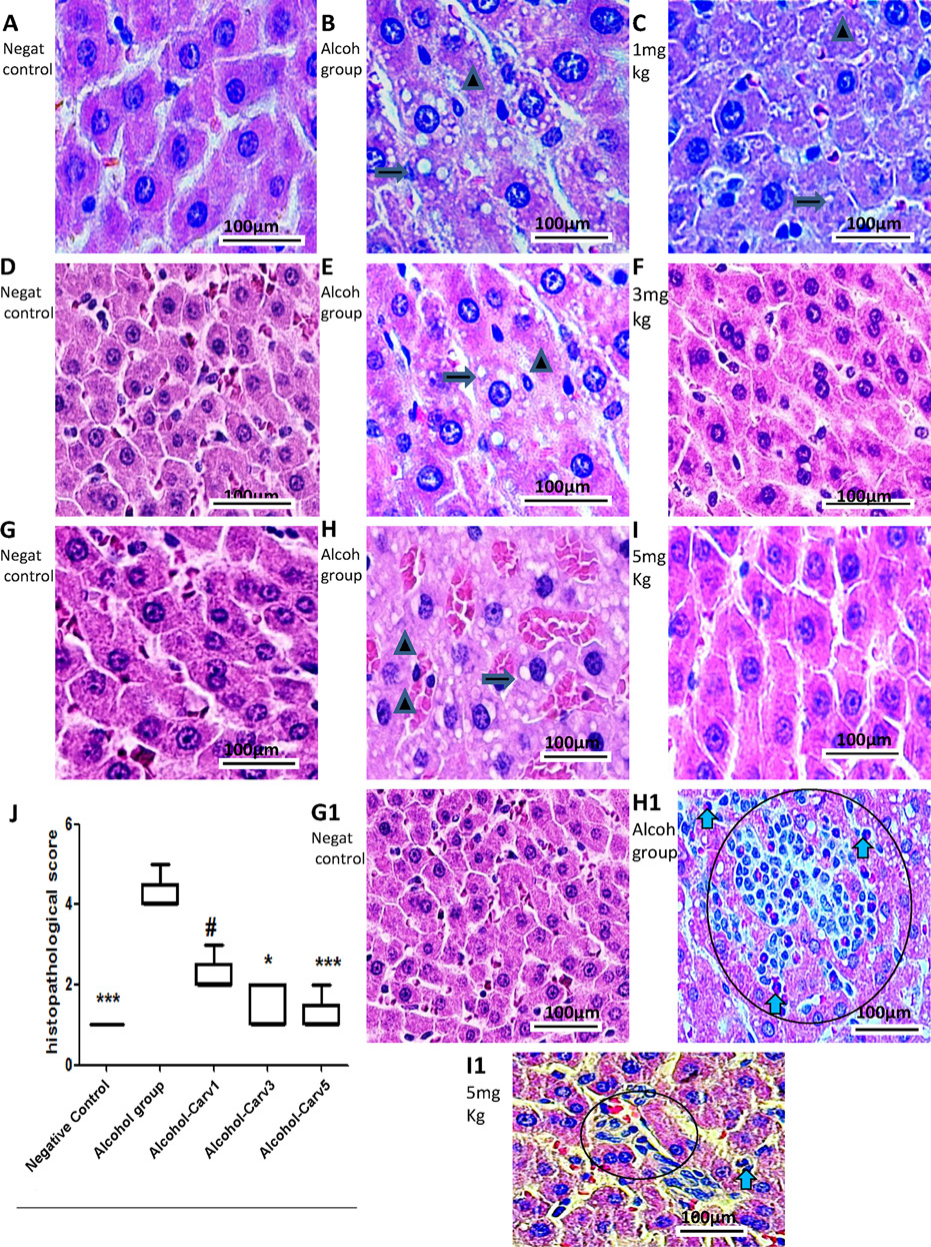
Histological examination of liver specimens in rats with alcohol-induced liver injury. Five animals per group and three H&E sections per animal were analysed. Images and data from the CARV 1 mg/ml, 3 mg/ml, and 5 mg/ml groups are shown in panels A–C, D–F, and G–I, respectively (arrow, fatty changes within hepatocytes;triangle, necrosis area). Negative control group slides are shown in A, D, and G. Alcohol group slides are shown in panels B, E, and H (middle images). In the alcohol-only group, rat livers exhibited steatosis with various degrees of diffuse hepatic steatosis and intralobular, deranged hepatic cord, and necrotic areas. Liver injury (*e*.*g*., steatosis and inflammation) persisted in the alcohol-CARV 1 mg/kg group (C). (F, I) Reduced steatosis and hepatocyte regeneration associated with reduced necrosis were observed in the livers of animals in the alcohol-CARV 3 mg/kg and alcohol-CARV 5 mg/kg groups. The neutrophils infiltrate the parenchyma, as seen here, and often surround ballooned hepatocytes (blue arrow: neutrophils and circle: lymphocytes and neutrophils infiltrate, Fig 3H1). Reduced neutrophilic infiltration was observed in the livers of animals in the alcohol-CARV 5 mg/kg groups (Fig 3I1). Magnification 400×, scale bar = 100 μm. (J) Representative samples from each CARV treatment group are shown with graphs summarizing the mean histopathological score of each group. (***p < 0.001 *vs*. negative control, #*p* > 0.05, *p < 0.05, and ***p < 0.001 *vs*. alcohol-only group).

There were no pathological changes observed after 28 days in the negative control animal livers, as indexed by a semiquantitative scoring system. Meanwhile, the livers from rats that were given alcohol for 28 days exhibited fat accumulation, inflammation, and necrosis, resulting in a high pathology scores (Fig 3J). Additionally, the alcohol-only treatment group had significantly greater steatosis than the negative control group (*p* < 0.001). Conversely, alcohol-induced liver damage was reduced in the alcohol-CARV 3 mg/kg and alcohol-CARV 5 mg/kg groups (p < 0.05 and p < 0.001 *vs*. alcohol-only group, respectively; Fig 3F, 3I and 3J), but not the alcohol-CARV 1 mg/kg group (*p* > 0.05 *vs*. alcohol-only group, Fig 3C and 3J). Reduced inflammation was most clearly observed in the alcohol-CARV 5 mg/kg group (Fig 3I1), which exhibited decreased areas of steatosis and reduced levels of necrosis relative to the alcohol-only group.

**Fig 4:**
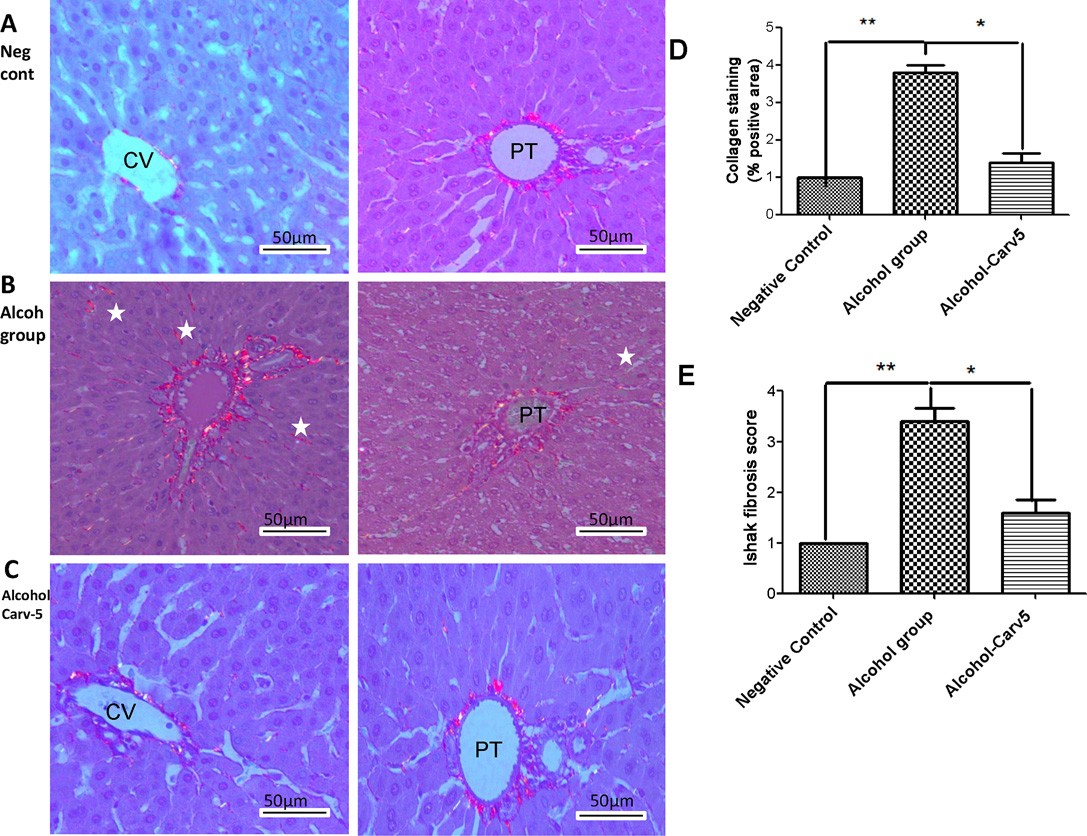
Representative photomicrographs of liver sections stained with picrosirius red. Five animals per group and three H&E sections per animal were analysed. (A) Negative control group livers had weak staining limited to centrilobular veins (left panel) and the portal tract (right panel). (B) Liver sections from the alcohol-only group exhibited marked portal fibrosis (left panel) and staining between the hepatic cords (right panel). (C) The alcohol-CARV 5 mg/kg group livers had a weak fibrotic response limited to centrilobular veins (left panel) and portal tract (right panel). (D) Morphometric quantification of Sirius red stained areas demonstrated an attenuation of the fibrotic process in the alcohol-CARV 5 mg/kg group compared to the alcohol-only group. (E) The area fraction of total fibrosis, including fibrosis in the portal tract area, in rats with alcohol-induced liver injury in relation to the Ishak score. The alcohol-CARV 5 mg/kg group livers had an attenuation of the fibrotic process. PT, portal tract; CV, central vein; asterisk, hepatic cords. Magnification 400×, scale bar = 50 μm. (**p < 0.01, *p < 0.05, Kruskal-Wallis test followed by Dunn’s test).

Livers from the alcohol-only group were characterized by excessive Sirius red-stained collagen I fibres (*p* < 0.05 *vs*. negative controls, Fig 4B). The stained fibres appeared orange-red in colour and were observed under polarized light, due to their increased thickness. Sirius red-stained collagen fibres were decreased in the alcohol-CARV 5 mg/kg to relative to alcohol-only group (*p* > 0.05, Fig 4C, 4D and 4E).

**Fig 5:**
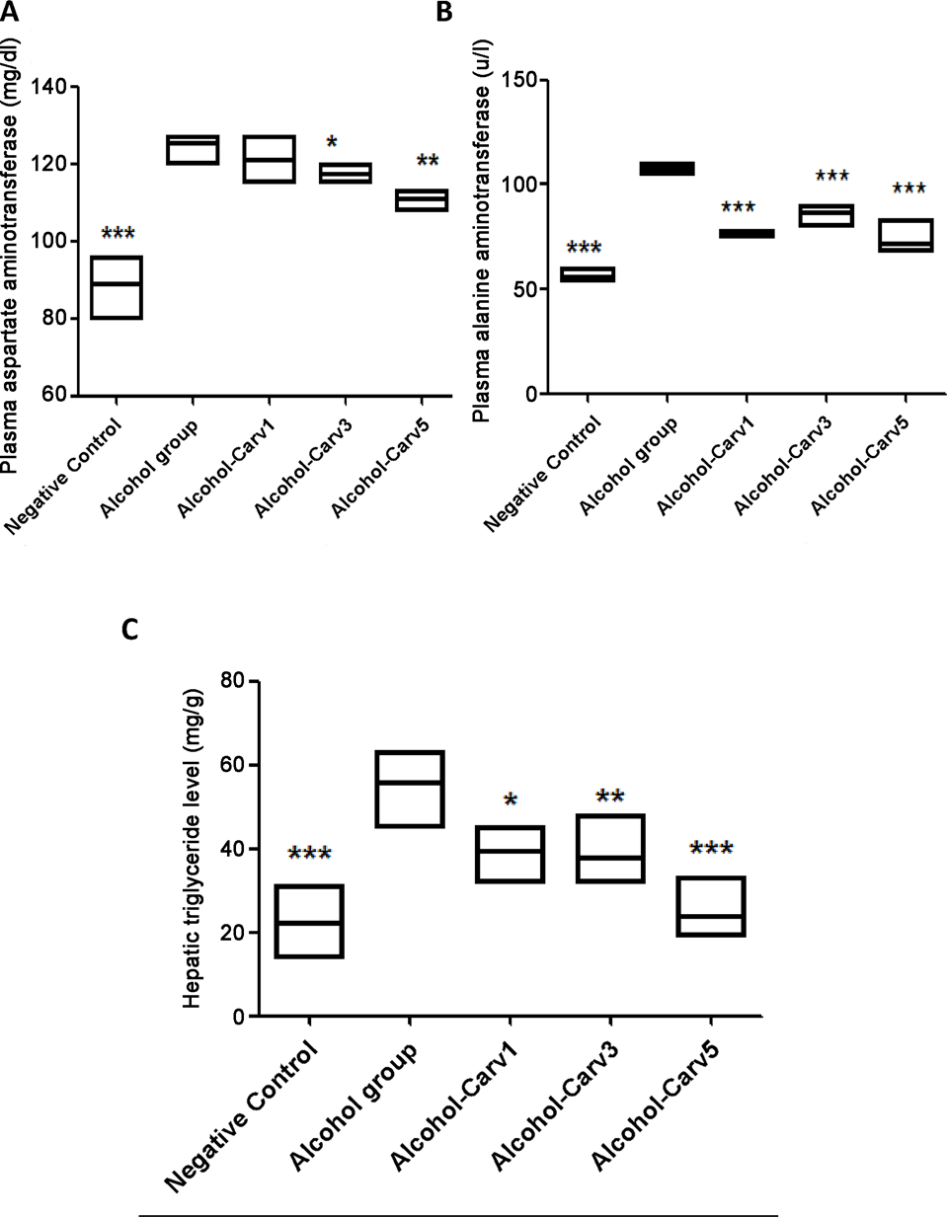
Biochemical analysis of blood AST (mg/dl), ALT (mg/dl), and triglyceride (mg/dl) levels in rats with alcohol-induced liver injury. CARV modified the ethanol-induced increases in levels of AST (A, n = 5, *p* < 0.01), ALT (B, n = 5, *p* < 0.01), and hepatic triglycerides (B, n = 5, *p* < 0.001). *p < 0.05, **p < 0.01, and ***p < 0.001 *vs*. the alcohol group and ***p < 0.001 for negative control *vs*. alcohol group (analysis of variance followed by Bonferroni’s correction).

H&E staining showed fatty droplet accumulation around the central vein (Fig 3B, 3E and 3H,) and the inflammatory infiltrate contains neutrophils and lymphocytes (Fig 3H1), with higher levels of AST (Fig 5A), ALT (Fig 5B) and hepatic triglycerides (TG) (Fig 5C) in livers from rats subjected to chronic alcohol exposure. However, CARV treatment modulated this alcohol-induced hepatosteatosis and liver injury (Fig 5).

### Immunohistochemistry and confocal immunofluorescence

**Fig 6:**
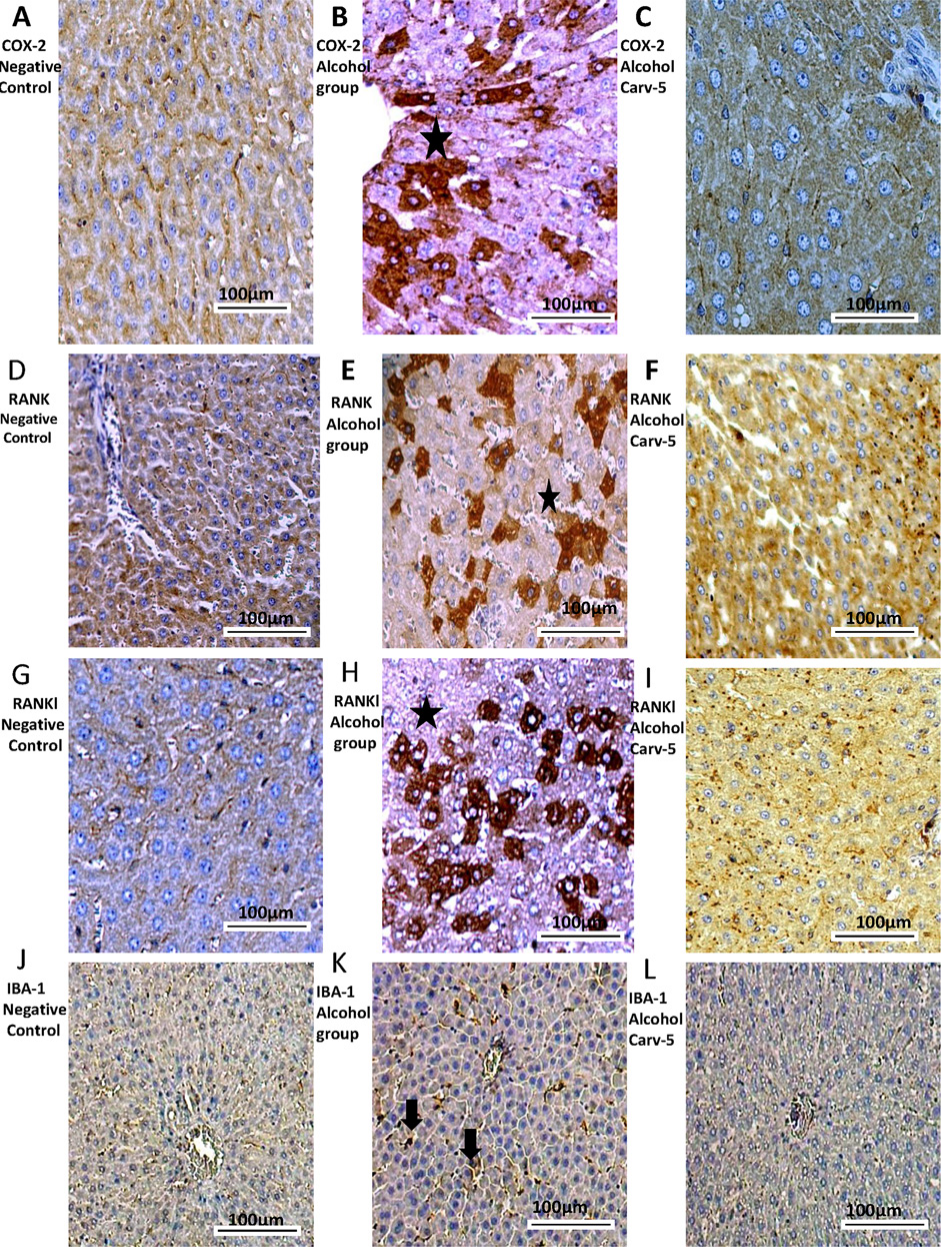
COX-2, RANK, RANK-L, and IBA-1 immunohistochemical findings. Generally, livers from alcoholic rats had greater COX-2 (B), RANK (E), RANK-L (H) and IBA-1 (K) immunoreactivity than livers from the negative control and alcohol-CARV 5 mg/kg groups. For each antigen, three immuno-labelled sections were analysed per animal (N = 5 animals per group). Asterisk: strong labelling; black narrow: labelling to Kupffer cells (IBA-1). Magnification 4000×, scale bar = 100 μm.

**Fig 7:**
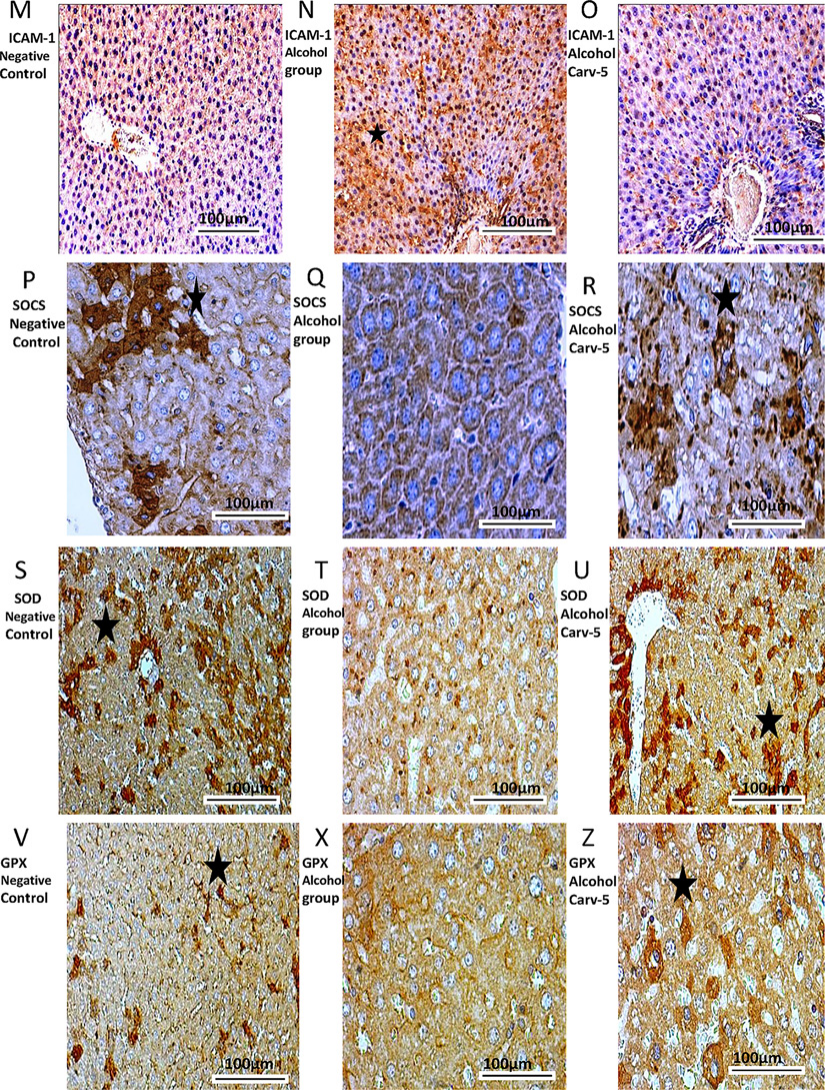
ICAM-1, SOCS1, SOD-1, and GPx immunohistochemical findings. Livers from alcohol-administered rats had greater ICAM-1 (N) immunoreactivity than the saline control and alcohol-CARV 5 mg/kg group rats. Livers from alcohol-administered rats had reduced SOCS1 (Q), SOD-1 (T), and GPx (X) immunoreactivity levels than the negative control and alcohol-CARV 5 mg/kg group rats. For each antigen, three immuno-labelled sections were analysed per animal (N = 5 animals per group). Asterisk: strong labelling. Magnification 4000×, scale bar = 100 μm.

**Fig 8:**
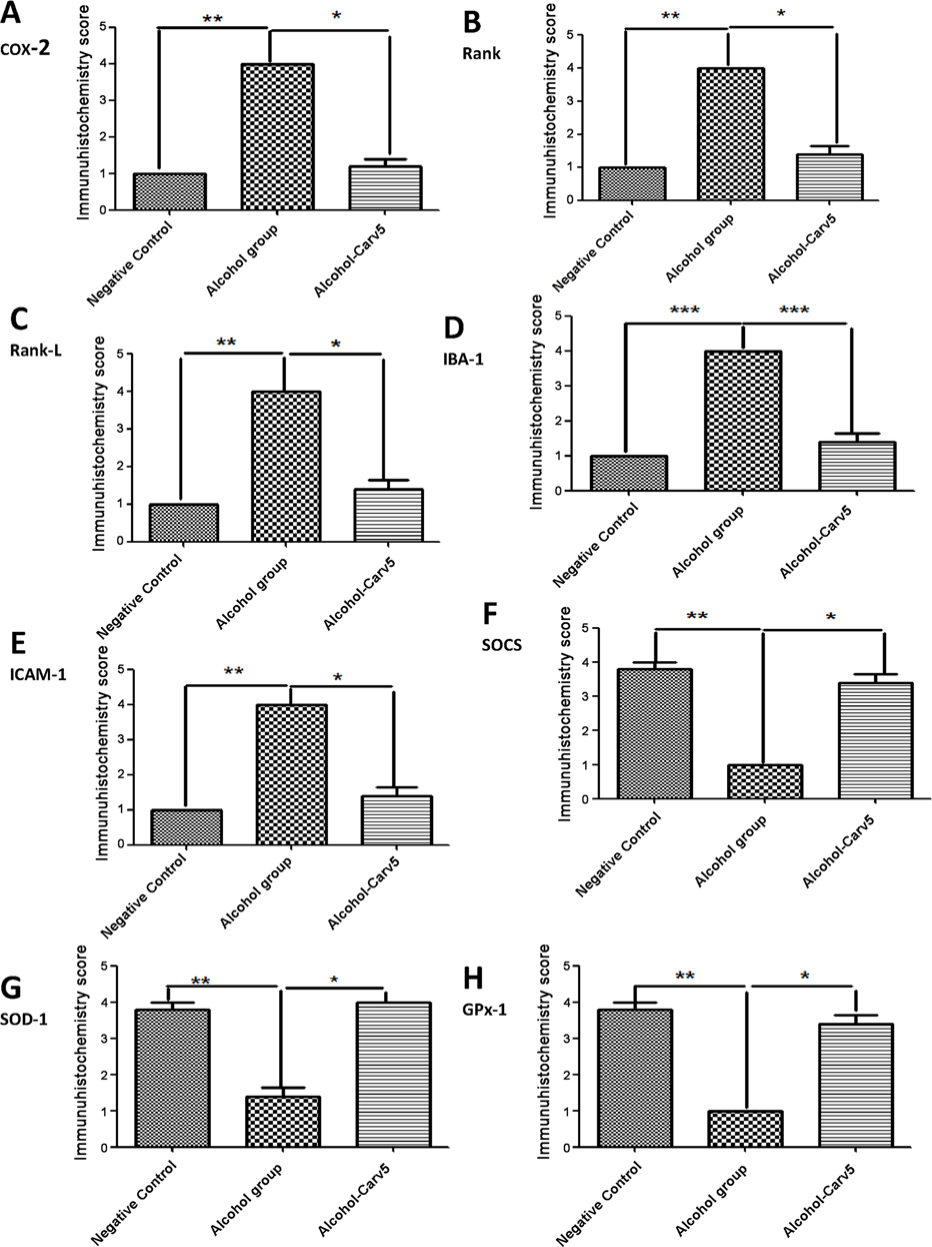
CARV effects on ethanol-induced liver injury in rats with alcohol-induced liver injury. Five immunohistochemistry sections from each animal in each group were analysed (N = 5 animals per group). Representative samples from the alcohol-CARV 5 mg/kg treatment group are shown with graphs summarizing the mean group scores for COX-2, RANK, RANK-L, IBA-1, ICAM-1, SOCs, SOD-1 and GPx-1 immunoreactivity. The alcohol-induced effects were reversed (*i*.*e*., normalized) in the alcohol-CARV 5 mg/kg livers. *p < 0.05 *vs*. alcohol group and ***p < 0.001 for negative control *vs*. alcohol group (Kruskal-Wallis test followed by Dunn’s test).

Compared to the alcohol-only group, the alcohol-CARV 5 mg/kg group exhibited reductions in levels of COX-2, RANK, RANK-L, IBA-1, and ICAM-1 (all *p* < 0.05; Fig 6C, 6F, 6I and 6L, Fig 7O and Fig 8A, 8B, 8C, 8D and 8E) and an increased levels of SOCS1, SOD-1, and GPx-1 (all *p* < 0.05, Fig 7R, 7U and 7Z and Fig 8F, 8G and 8H). These changes were consistent with the normalization toward non-alcohol exposure levels.

**Fig 9:**
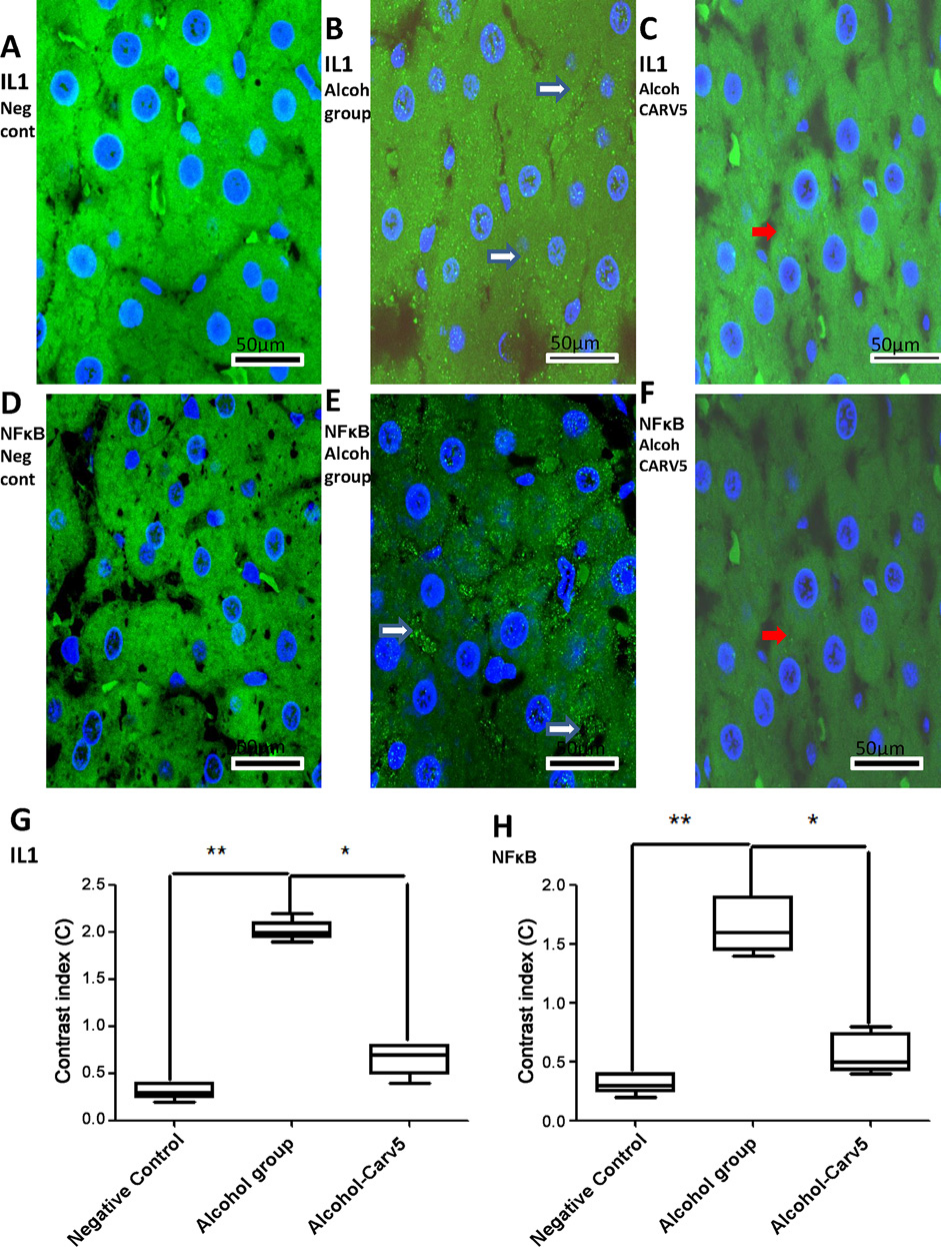
CARV modulates IL-1β and NF-κB expression. Representative confocal photomicrographs of IL-1β and NF-κB immunoreactivity in liver specimens from each group (green) with DAPI nuclear counterstained (blue). (A,D) Negative control rat liver without IL-1β and NF-κB labelling, respectively. (B,E) IL-1β and NF-κB labelling (white narrow) was diffuse and strong in the alcohol-only group, respectively. Besides, the NF-κB labelling was more concentrated between the hepatic cell cords. (C,F) Weak IL-1β and NF-κB labelling (red arrows) was seen in the alcohol-CARV 5 mg/kg group, respectively. Scale bar, 50 mm. (G,H) Densitometric analysis confirmed significant increases in IL-1β and NF-κB immunoreactivity in the alcohol-only group that were blocked in the alcohol-CARV 5 mg/kg group. Five immunofluorescence sections from each animal in each group were analysed (N = 5 animals per group) (**p < 0.01, *p < 0.05, Kruskal-Wallis test followed by Dunn’s test).

Cellular IL-1β and NF-κB labelling (green) were strong and diffuse in the alcohol group (Fig 9B and 9E), weak in the alcohol-CARV 5 mg/kg group (Fig 9C and 9F), and absent in the negative control group (Fig 9A and 9D). Densitometric analysis confirmed that there were significantly increased IL-1α and NF-κB immunoreactivities in the alcohol-only group, relative to the negative control group, that were blocked in the alcohol-CARV 5 mg/kg group (Fig 9G and 9H).

### CARV treatment decreased mRNA expression of TNFα, PCI, PCIII and NF-κB

**Fig 10:**
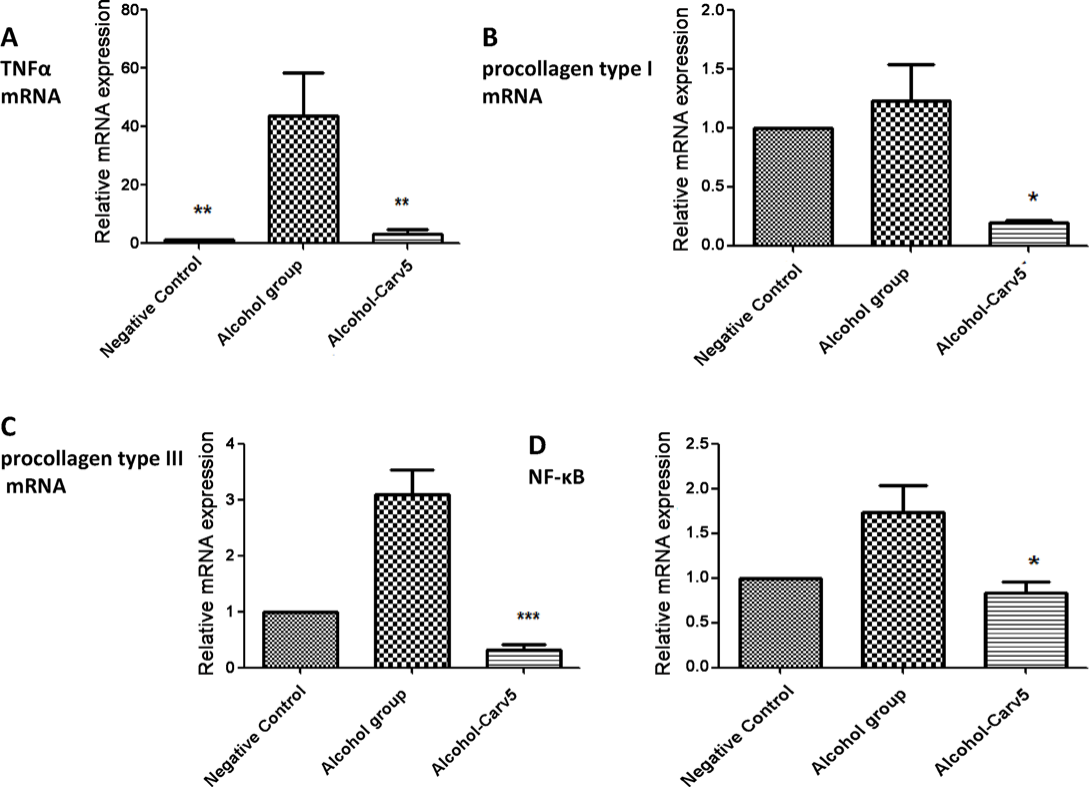
CARV effects on TNFα, PCI, PCIII, and NF-κB mRNA expression in rats with alcohol-induced liver injury. The expression of TNFα mRNA was increased in alcohol group (**p< 0.01, Fig 10A) and decreased in alcohol- CARV 5 mg/kg group (**p< 0.01, Fig 10A). The expression of PCI mRNA was decreased in alcohol- CARV 5 mg/kg group compared to the alcohol-only group (*p< 0.05, Fig 10B). PCIII mRNA levels appeared to be lower in the alcohol-CARV 5 mg/kg group compared to the alcohol-only group (***p<0.001, Fig 10C) and the expression of NF-κB mRNA was decreased in alcohol- CARV 5 mg/kg group compared to the alcohol-only group (*p>0.05, Fig 10D). (N = 5 animals per group; Kruskal-Wallis test followed by Dunn’s test).

TNFα mRNA expression was significantly decreased in the alcohol-CARV 5 mg/kg group relative to levels in the alcohol-only group (*p* < 0.01, Fig 10A). The expression of PCI and PCIII mRNA was decreased in alcohol- CARV 5 mg/kg group compared to the alcohol-only group (*p* < 0.05 and p<0.001, respectively, Fig 10B and 10C). In addition to this, the expression of NF-κB mRNA was decreased in alcohol- CARV 5 mg/kg group compared to the alcohol-only group (p>0.05, Fig 10D).

## Discussion

In the present study, we observed severe fat accumulation, mild inflammation, necrosis and accumulation of PCI and PCIII around centrilobular hepatic vein and the hepatic triad, resulting in moderate steatosis, as well as elevated neutrophil, MPO, and pro-inflammatory cytokine levels in animals given alcohol (by gavage) for 4 weeks. Meanwhile no pathological changes, neutrophil infiltration, MPO, or cytokine level changes were observed over the same time period in saline control rats. Moreover, the alcohol-related changes were dampened significantly in animals in the alcohol-CARV 5 mg/kg group.

Despite major advances in our understanding of the pathogenesis of alcohol-related liver injury, there are no Food and Drug Administration-approved treatments for ALD. Treatment of the underlying addiction, aggressive nutritional intervention, and “off-label” use of various pharmacotherapies aimed at the underlying mechanisms of injury (*e*.*g*., cytokine dysregulation, endotoxin translocation, and oxidative stress) represent the primary approaches to treating ALD.

Macrovesicular steatosis—the first and most common pathologic change associated with chronic alcohol ingestion—is observed in up to 90% of heavy alcohol users [25]. These changes are reversed by eliminating alcohol consumption. Hepatocyte apoptosis and areas of microvesicular steatosis can also be seen in livers with chronic alcohol exposure, although they are more common in steatohepatitis (a.k.a. alcoholic hepatitis). Ballooning degeneration of hepatocytes, infiltrating neutrophils, Mallory bodies, and fibrosis are pathologic findings indicative of steatohepatitis [26].

Many studies have implicated proinflammatory cytokines, especially TNF-α, in the development and progression of ALD. Several clinical manifestations of ALD resemble the biological effects observed after *in vivo* administration of proinflammatory cytokines, suggesting that cytokines contribute to clinical complications and liver injury [27]. Administration of TNF-α antibodies prevented liver necrosis and inflammation, but not steatosis, in rats [28] and knockout mice lacking TNF receptor 1 were protected from the inflammatory responses associated with chronic ethanol administration. These findings suggest strongly that TNF-α may be a proximal mediator of ethanol-induced liver damage.

Additionally, the proinflammatory cytokines IL-1 and IL-6 and the chemokine IL-8 are important mediators of ALD. Each may enhance the effects of the others. For example, although IL-1 participates in the pathogenesis of ALD, it does not seem to cause liver injury alone; rather, it appears to act synergistically with TNF-α [29]. Our present findings showing that CARV treatment decreased alcohol-induced changes in IL-1β and TNF-α levels support the notion that these factors are involved in the pathogenesis of ALD.

CARV’s anti-inflammatory influence has been associated with reductions in TNF-α and IL-1β levels coincident with increased IL-10 levels [30]. IL-10 is a potent anti-inflammatory molecule that has been shown to inhibit the production of TNF-α and IL-1 and to suppress the activation of NF-κB [31]. IL-10 reduces macrophage production of nitric oxide and reactive oxygen intermediates, and also reduces the expression of adhesion molecules and chemokines [32].

Our present observation of increased MPO activity, suggestive of neutrophil infiltration, in alcohol-injured livers is consistent with previous observations. For example, Jan Petrasek *et al*. (2010) demonstrated that alcoholic steatohepatitis is characterized by infiltration of various inflammatory cells, including monocytes, macrophages, neutrophils, and lymphocytes, as a consequence of the activation of inflammatory mediators induced by Toll-like receptors signalling [33]. Neutrophils that accumulate in the hepatic microvasculature (sinusoids and postsinusoidal venules) can extravasate/transmigrate into the hepatic parenchyma after receiving signals from distressed cells. Neutrophils adhere to the distressed hepatocytes through neutrophil β_2_ integrins and hepatocyte ICAM-1. ICAM-1—a member of the immunoglobulin superfamily and critical adhesion molecule expressed on several cell types, including endothelial cells, epithelial cells, and fibroblasts—is induced by pro-inflammatory cytokines, including TNF-α and IL-1 [34]. Neutrophil contact with hepatocytes mediates the oxidative killing of hepatocytes by initiation of the respiratory burst and neutrophil degranulation, leading to hepatocellular oncotic necrosis. Neutrophil-mediated liver injury has been demonstrated in various diseases and chemical/drug-induced toxicities [31].

Our present findings that MDA levels were increased in the alcohol-only group, and that this increase was blocked in the alcohol-CARV 5 mg/kg group, are consistent with prior research showing that increases in MDA are found in chronic-alcoholism model rats and human alcoholics [35]. The lipid peroxide MDA, a widely used indirect biomarker of oxidative stress, is generated when free radicals produced by chronic, excess ethanol exposure attack polyunsaturated fatty acids in membranes. In the intragastric ethanol infusion rodent model, liver damage is associated with enhanced lipid peroxidation, decreased formation of carbonyl in GSH, and formation of 1-hydroxyethyl radicals and lipid radicals [36]. Reactive aldehydes, such as acetaldehyde, are produced from ethanol metabolism and ethanol-induced oxidant stress.

Ethanol administration can also generate ethanol-derived free radicals via at least two pathways: production of hydroxyl radicals from endogenous H_2_O_2_ in a Fenton-type reaction; and CYP2E1-mediated radical generation. The resultant ethanol-derived free radicals can exacerbate reactive oxygen species (ROS)-mediated cellular damage [37]. The present findings suggest that CARV (5 mg/kg) can counter these effects by increasing levels of both enzymatic (*i*.*e*. SOD and GPx-1) and non-enzymatic (*i*.*e*. GSH) antioxidant defences. These results are consistent with prior research showing that CARV protects cells against acetaminophen-induced oxidative injury and suggesting that CARV’s cytoprotective effects may be related the O_2_^-^ scavenging properties of CARV or its metabolites [38].

Hepatocytes subjected to inflammatory conditions release IL-1β and TNFα, which leads to NF-κB activation and inflammation [39]. Chemokines and cytokines are involved in the pathogenesis of alcoholic hepatitis and contribute to leukocyte migration into the liver during chronic ethanol intoxication. These compounds are associated with increased basal H_2_O_2_ formation and enhanced activation of NF-κB in Kupffer cells. Chronic alcoholic injury augments the activation of NF-κB and the production of proinflammatory cytokines, including TNF-α and IL-1 [40]. Our results showing that CARV at the 5 mg/kg dosage significantly inhibited alcohol-induced IL-1β and TNFα levels and the mRNA expression of NF-κB supports the notion that reduces inflammatory conditions.

COX-2–deficient mice are protected against the toxic effects of endotoxin [41]. Expression of COX-2 is increased in alcoholic liver injury, in association with necro-inflammatory changes and endotoxemia. Kupffer cells (recognized by their IBA-1 expression) are the primary source of COX-2 in the liver [42]. The present results showing that alcohol-induced increases in COX-2, IBA-1, and ICAM-1 expression levels were countered by CARV treatment (5 mg/ml) suggest that CARV may block the activation of Kupffer cells, thereby preventing the subsequent production of TNF-α and COX-2.

SOCS1 is an important suppressor of cytokine signalling and inflammation, suppressing the activation of many cytokines, including IL-2, IL-3, IL-4, IL-6, interferon (IFN)-α, IFN-β, IFN-γ, and TNF-α [43,44]. SOCS1-mediated suppression of inflammation involves regulatory effects on innate immune cells and nonimmune cells. Selective hepatocyte depletion of SOCS1 in liver-specific SOCS1 knockout mice resulted in enhanced concanavalin A-induced hepatitis associated with pro-apoptotic signals (including STAT1 and JNK) [45]. Cytoplasmic SOCS1 has been hypothesized to regulate the transcription factor NF-κB [46,47]. Interestingly, in this study, the anti-inflammatory influence of CARV was accompanied by an increase in RANK/RANKL expression and elevated SOCS1 expression levels, suggesting that CARV-induced increases in RANK/RANKL complexes may prevent the nuclear translocation of NF-κB and, thereby, reduce the production of proinflammatory cytokines [27].

Alcoholic liver fibrosis is characterized by excessive deposition of ECM proteins, especially collagen types I and III. Activated HSCs produce large amounts of ECM components rapidly, triggering a fibrogenic response [11]. Before liver fibrosis occurs, hepatocellular damage is initiated during the cross-talk between liver cell types mediated by cytokines, ROS, and other soluble factors. Our findings show that CARV (5 mg/kg) treatment inhibited the mRNA expression of PCI and PCIII significantly is consistent with the notion that CARV may counter alcohol-induced changes in HSC biosynthesis.

On the basis of our findings, we hypothesise that the protective effects of CARV treatment against alcoholic liver injury are mediated through suppression of inflammatory cytokine and ROS induction. We suggest that CARV reduces inflammation and oxidative stress in the liver caused by alcohol, thereby reducing the activity of HSCs and Kupffer cells. The present work extends prior research demonstrating the antioxidant activity of CARV in alcohol-induced liver injury [15] and the preventive role of CARV on the development of hepatosteatosis [17] by providing information about molecular changes in inflammatory pathways.

In summary, inhibition of the SOCS1 signalling pathway by alcohol was reversed by CARV treatment, resulting in liver recovery. Specifically, CARV treatment reduced the expression levels of IL-1β and TNF-α, downregulated expression of COX-2, RANKL/RANK, IBA-1 and ICAM-1, and upregulated SOCS1, SOD-1 and GPx-1 expression. Additionally, CARV treatment inhibited hepatic expression of several pro-inflammatory mediators and ROS levels in ethanol-fed rats by modulating the activity of Kupffer cells and HSCs. The positive effect of CARV on liver architecture in alcoholic liver injury suggests that CARV treatment may aid in the recovery of hepatosteatosis, been sufficient to prevent liver fibrosis.
